# Is the nucleus the unwitting architect of asymmetric cell division in plants?

**DOI:** 10.1093/plphys/kiad379

**Published:** 2023-07-03

**Authors:** Janlo M Robil

**Affiliations:** Assistant Features Editor, Plant Physiology, American Society of Plant Biologists, Rockville, MD 20855, USA; Department of Biology, School of Science and Engineering, Ateneo de Manila University, Quezon City 1108, Philippines

Generation of diverse cell types and their assembly into complex tissues are fundamental to multicellular development. In plants, this process is a unique challenge because cell movement is restricted by cell walls. To overcome this challenge, plants use asymmetric cell division as a carpentry trick to build complex tissues, producing cells with distinct fates that are often of unidentical size and shape. One of the most elegant demonstrations of this process is observed in the formation of stomata, the plant's “breathing” pores. Asymmetric cell division facilitates the generation and patterning of guard cells (GCs) and the accompanying subsidiary cells (SCs). Over the past 2 decades, studies have drawn the blueprints for stomatal development in Arabidopsis and grasses, revealing mechanisms controlling asymmetric cell division (Guo et al. 2021). Yet an overarching question remains: how do the cells determine the division site?

Multiple factors, including cell polarity cues and the cell division machinery, contribute to determining the future site of division in asymmetrically dividing cells. However, it is not yet known whether the nucleus itself plays a role. In this issue of *Plant Physiology*, Ashraf et al. (2023) address that question. Using maize mutants defective in the outer nuclear membrane protein MAIZE LINC KASH SINE-LIKE2 (MLKS2) (Gumber et al. 2019), the authors provide genetic and cellular evidence that nuclear positioning plays a role in the specification of the division plane during stomatal development.

**Figure 1.**
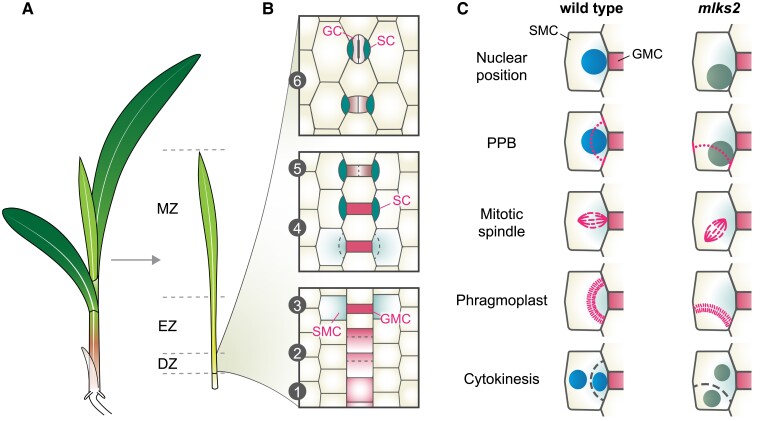
Developmental trajectory of maize stomata and MLKS2-mediated nuclear positioning and division plane specification. **A)** A maize seedling showing the developmental gradient of an emerging leaf. **B)** Schematic representation of stomata development at the base of the leaf: (1) the stomatal file is specified from undifferentiated epidermal cells; (2) the cells in the stomatal file undergo transverse asymmetric division (dashed lines) to produce the GMCs and the interstomatal cells; (3) SMCs are recruited from neighboring lateral cells and (4) they undergo longitudinal asymmetric division (dashed lines) to produce the SCs and the lateral pavement cells; (5) the GMC undergoes longitudinal symmetric division to produce the GCs; (6) the GCs and SCs differentiate to form the stomata. **C)** Schematic representation of nuclear positioning and cell division structures in wild-type and *mlks2* SMCs. Ashraf et al. (2023) found that the mispositioned nucleus in *mlks2* SMCs correlates with misplaced PPB (dotted line) and incorrect division plane during cytokinesis (dashed line), indicating a role of nuclear positioning in cell division site specification. DZ, division zone; EZ, elongation zone; MZ, maturation zone.

First, the authors characterized and quantified the stomatal development defects in *mlks2* mutants. Unlike the typical kidney-shaped GCs in eudicots, stomata in maize and other grasses consist of elongated dumbbell-shaped GCs flanked laterally by 2 lens-shaped SCs (Nunes et al. 2020). The guard mother cells (GMCs) are formed by transverse asymmetric cell division in the stomatal file at the base of developing leaves. The GMCs recruit the subsidiary mother cells (SMCs) from neighboring lateral cells, which asymmetrically divide to produce the SCs and lateral pavement cells. Finally, the GMCs undergo longitudinal symmetric division to form the GCs (Fig. 1, A and B). The authors found aberrant stomatal patterns in *mlks2* mutants, which point to asymmetric division defects during GC and SC formation. To determine the cellular basis for these defects, the authors performed a series of confocal microscopy analyses, focusing on the subcellular organization and behavior of the SMCs before and during asymmetric division (Fig. 1C).

The establishment of cell polarity is required for correct asymmetric division in the SMCs, and this is followed by the migration of the nucleus toward the GMC (Gallagher and Smith 2000; Facette et al. 2015). The authors found normal subcellular localization of specific cell polarity markers in the mutant. These findings suggest that the defects in *mlks2* are not due to cell polarity and could be associated with nuclear movement, consistent with the proposed role of the protein (Gumber et al. 2019). To test this idea, the authors examined nuclear positioning in the SMCs and found that a significantly higher number of nuclei failed to migrate toward the GMC in *mlks2.* Therefore, Ashraf et al. (2023) provide evidence that MLKS2 is required for proper nuclear positioning independent of cell polarity.

The authors hypothesized that the abnormal nuclear positioning in *mlks2* SMCs affects the orientation of the division plane, which is initially marked by the appearance of the preprophase band (PPB). To test this hypothesis, they examined nuclear positioning in SMCs with a visible PPB. They found that there was a higher incidence of misoriented PPBs in *mlks2* and that more cells in the mutant showed both misoriented PPB and mispositioned nucleus. Next, the authors examined whether mitotic spindle orientation is affected in *mlks2* SMCs. They found that mutant cells displayed significantly higher variation in the mitotic spindle angle. Additionally, cells with abnormal cortical division sites exhibited more extreme spindle angles. These *mlks2* phenotypes raise the question of whether the nucleus coordinates PPB formation and spindle orientation. This question is important because the role and significance of these events in directing plant cell division remain unresolved (Yi and Goshima 2022).

Ashraf et al. (2023) used time-lapse imaging to show that PPB and nuclear positions determine spindle and phragmoplast positions in dividing SMCs. In wild-type cells, the nuclei correctly positioned next to the GMC, the PPBs formed normally, and the spindle and phragmoplast formed perpendicular to each other at the expected division site. By contrast, over 25% of *mlks2* cells had nuclei that failed to position correctly and PPBs that formed at the wrong sites. In these cells, the spindle and phragmoplasts formed at an oblique angle, consistent with the locations of the PPBs (Fig. 1C). These data suggest that the aberrant division plane orientation in *mlks2* follows the misplaced PPB and mispositioned nucleus.

To determine why *mlks2* nuclei are mispositioned, the authors used time-lapse imaging to track nuclear movement in SMCs before and after division plane specification. They found that, in addition to the abnormal pre-mitotic movement, *mlks2* nuclei failed to maintain their position after division plane specification. Taken together, these findings suggest that MLKS2 is important for both nuclear migration and stable nuclear positioning, which are necessary for PPB placement and division plane specification during asymmetric cell division of the SMCs.

The work of Ashraf et al. (2023) advances our understanding of asymmetric cell division in plants. It supports the hypothesis that the nucleus plays a crucial role in determining the site of cell division, and further research is needed to comprehend how the nuclei affect cytoskeletal mechanisms in various cell types, including those without PPBs (Yi and Goshima 2022). The meticulous experiments presented in this study lay the groundwork for future research, which could benefit from advancements in object and single-particle tracking techniques and computational modeling. The study highlights the significance of nuclear movement in cell fate specification and could have implications for the understanding of tissue and organ patterning. As such, the discovery in this study could contribute to designing a more efficient stomata arrangement or density, which will be critical for engineering climate-resilient grass crops (Nunes et al. 2020).
